# Social–Cognitive Factors in Antisocial Behavior and School Violence: A Cross-Sectional Analysis of Greek Vocational Students

**DOI:** 10.3390/children12121647

**Published:** 2025-12-04

**Authors:** Anastasia Petropoulou, Hera Antonopoulou, Agathi Alexandra Vlachou, Evgenia Gkintoni, Constantinos Halkiopoulos

**Affiliations:** 1 Department of Management Science and Technology, University of Patras, 265 04 Patras, Greece; b12petr@ionio.gr; 2 Department of Medicine, Aristotle University of Thessaloniki, 541 24 Thessaloniki, Greece; avlachoy@gmail.com; 3 Department of Psychiatry, University General Hospital of Patras, 265 04 Patras, Greece; evigintoni@upatras.gr

**Keywords:** school violence, antisocial behavior, social–cognitive factors, aggression norms, vocational education, teacher–student relationships, adolescent attitudes, Greek students

## Abstract

**Background/Objectives:** School violence represents a significant concern for educational communities worldwide, affecting student well-being and academic development. While prior research has documented prevalence rates and risk factors, limited studies have examined social–cognitive factors associated with antisocial behavior specifically within vocational education contexts using integrated analytical approaches. This exploratory cross-sectional study examined social–cognitive factors—specifically self-reported attitudes about aggression norms, prosocial attitudes, and school climate perceptions—associated with violence-supportive attitudes among Greek vocational students. **Methods:** A cross-sectional design employed validated self-report instruments and traditional statistical methods. The sample comprised 76 vocational high school students (38.2% male; ages 14–18; response rate 75.2%) from one school in Patras, Greece. Validated instruments assessed attitudes toward interpersonal peer violence (α = 0.87), peer aggression norms across four subscales (α = 0.83–0.90), and school climate dimensions (α = 0.70–0.75). Analyses included descriptive statistics, Pearson correlations with bootstrapped confidence intervals, MANOVA for multivariate group comparisons, independent samples *t*-tests, propensity score matching for urban–rural comparisons, polynomial regression for developmental patterns, and path analysis for theoretical model testing. **Results:** Strong associations emerged between perceived school-level and individual-level aggression norms (r = 0.80, *p* < 0.001, 95% CI [0.71, 0.87]), representing one of the strongest relationships documented in school violence research. Violence-supportive attitudes demonstrated inverse associations with prosocial alternative norms (r = −0.37, *p* < 0.001, 95% CI [−0.55, −0.16]). Significant gender differences emerged for teacher–student relationships (d = −0.78, *p* = 0.002), with females reporting substantially more positive perceptions. Propensity-matched urban students demonstrated higher aggression norm endorsement compared to rural students across multiple indicators (d = 0.61–0.78, all *p* < 0.020). Polynomial regression revealed curvilinear developmental patterns with optimal teacher relationship quality during mid-adolescence (ages 15–16). Path analysis supported a sequential association model wherein school-level norms related to individual attitudes through prosocial alternative beliefs (indirect effect β = −0.22, *p* = 0.002, 95% CI [−0.34, −0.11]). **Conclusions:** This preliminary investigation identified social–cognitive factors—particularly normative beliefs about aggression at both individual and environmental levels—as strongly associated with violence-supportive attitudes in Greek vocational education. The exceptionally strong alignment between school-level and individual-level aggression norms (r = 0.80) suggests that environmental normative contexts may play a more substantial role in attitude formation than previously recognized in this educational setting. Gender and urban–rural differences indicate meaningful heterogeneity requiring differentiated approaches. Future research should employ longitudinal designs with multi-informant assessment and larger multi-site samples to establish temporal precedence, reduce method variance, and test causal hypotheses regarding relationships between normative beliefs and behavioral outcomes.

## 1. Introduction

School violence represents a significant concern for educational communities worldwide, affecting student well-being and academic development [1,2,3]. The phenomenon manifests across diverse educational settings, with documented negative impacts on academic performance, psychological health, and social development [1,4,5]. In Greece, the prevalence of school violence has been documented across educational settings [6,7,8,9,10], with vocational education students potentially facing unique risk factors related to educational trajectories, peer composition, and institutional characteristics [11,12]. Understanding the social–cognitive factors associated with violence-supportive attitudes among adolescents in vocational education contexts requires examination of normative beliefs, attitudinal patterns, and environmental influences that may contribute to aggressive behavior [13,14,15,16].

### 1.1. The Social–Cognitive Attitudinal Framework of Antisocial Behavior

Social–cognitive theory provides a comprehensive framework for understanding how individuals develop, maintain, and potentially modify attitudes toward aggression and violence [17]. This perspective emphasizes that behavior emerges from reciprocal interactions among cognitive processes, environmental influences, and behavioral patterns [17,18]. Within educational contexts, students develop attitudes about the acceptability, utility, and consequences of aggressive behavior through observational learning, direct experience, and normative feedback from peers and adults [19,20].

The current study focuses specifically on measurable attitudinal manifestations of these social–cognitive processes through validated self-report instruments, rather than objective neuropsychological assessment. We assess conscious beliefs, normative perceptions, and attitudinal orientations—the social–cognitive factors accessible through questionnaire methodology—rather than objective neuropsychological performance or executive function testing [21,22,23,24,25]. This approach aligns with established social–cognitive research traditions examining how attitudes and beliefs relate to behavioral outcomes in educational settings [17,19], recognizing that self-reported attitudes represent important proximal influences on behavioral choices even when neuropsychological capacities remain unmeasured [26,27].

#### 1.1.1. Attitudes About Self-Control

Self-control attitudes—beliefs about one’s capacity to regulate impulses, delay gratification, and inhibit aggressive responses—represent central components of social–cognitive frameworks [28]. Students who perceive themselves as capable of behavioral regulation demonstrate lower engagement in aggressive acts, while those reporting difficulty with impulse management show elevated violence risk [29,30]. These self-perceptions develop through accumulated experiences of successful or unsuccessful behavioral regulation across social contexts [31,32]. Research demonstrates associations between executive function capacities and antisocial outcomes, with deficits in inhibitory control, planning, and working memory relating to elevated aggression risk [33,34,35]. However, individual differences in self-perceived regulatory capacity—independent of objective performance—also predict behavioral outcomes [28,30]. In vocational education settings, where students may have experienced academic difficulties or behavioral challenges in traditional education, self-control attitudes may reflect historical patterns of regulatory success or failure that influence current behavioral choices [12,36].

#### 1.1.2. Social Information Processing Patterns

Social information processing theory describes how individuals encode, interpret, and respond to social cues in potentially conflictual situations [20]. Students who attribute hostile intent to ambiguous social interactions, generate primarily aggressive response options, or evaluate aggressive responses positively demonstrate elevated violence risk [37,38]. These cognitive patterns operate largely automatically, shaped by accumulated social experiences and reinforcement histories [20,39]. Research demonstrates that adolescents vary substantially in how they process social information, with some individuals showing systematic biases toward aggressive interpretations and responses [19,20,40]. Early life adversity, including maltreatment and trauma exposure, can alter social information processing pathways, increasing hostile attribution biases and aggressive response generation [41,42,43]. In vocational education contexts, peer composition and school climate may influence which information processing patterns receive social reinforcement [44,45].

#### 1.1.3. Empathic Attitudes and Perspective-Taking

Empathic attitudes—the capacity and willingness to consider others’ emotional experiences and perspectives—serve protective functions against aggressive behavior [46,47,48]. Students who readily engage in perspective-taking and experience emotional resonance with others’ distress show reduced violence involvement [49,50]. These empathic orientations develop through modeling, direct instruction, and experiences that encourage consideration of others’ viewpoints [51]. Individual differences in empathic attitudes emerge early in development and show relative stability, though educational interventions can enhance empathic capacities [50,52,53]. Research indicates that empathy operates through multiple pathways—cognitive empathy (understanding others’ perspectives) and affective empathy (emotional resonance with others’ feelings)—with differential associations to prosocial and antisocial outcomes [48,51]. Vocational education settings may provide particular opportunities for empathy development through collaborative project-based learning and diverse peer interactions, though they may also contain peer norms that discourage empathic expression [54,55].

#### 1.1.4. Normative Beliefs About Aggression

Normative beliefs—perceptions about the acceptability and prevalence of aggressive behavior within one’s social environment—demonstrate particularly strong associations with violent behavior [56,57,58]. Students who perceive aggression as normative, acceptable, and prevalent within their peer groups show substantially elevated violence risk compared to those perceiving aggression as exceptional and unacceptable [54,59]. These normative beliefs operate at multiple levels: individual beliefs about personal acceptability of aggression, perceptions of peer group norms, and perceptions of broader school-level normative climates [44,45,60]. Research indicates that perceived norms often exert stronger behavioral influence than actual base rates of aggressive behavior, suggesting that subjective perceptions rather than objective prevalence drive norm-behavior associations [45,60].

The alignment between individual normative beliefs and perceived environmental norms represents a critical research question. Strong correspondence might indicate accurate environmental perception, social influence processes wherein environmental norms shape individual beliefs, selection effects wherein individuals with similar beliefs cluster together, or projection wherein individual beliefs bias environmental perceptions [44,60,61]. Distinguishing among these mechanisms requires longitudinal and experimental designs, though cross-sectional associations provide preliminary evidence of norm–attitude relationships warranting further investigation [62,63].

### 1.2. Developmental Trajectories During Adolescence

Adolescence (ages 10–18) represents a developmental period characterized by substantial changes in social cognition, peer relationships, and behavioral regulation [64,65,66]. Early adolescence (ages 10–13) involves initial navigation of complex peer hierarchies, emerging autonomy from parental supervision, and developing capacity for abstract social reasoning [67,68]. Middle adolescence (ages 14–16) typically shows peak sensitivity to peer influence, consolidation of identity, and refinement of perspective-taking abilities [64,69]. Late adolescence (ages 17–18) involves preparation for adult roles, decreased peer conformity pressure, and crystallization of value systems [70,71].

Violence involvement typically shows developmental patterns with onset during early-to-middle adolescence, potential escalation during middle adolescence when peer influence peaks, and desistance for many individuals during late adolescence as adult roles and responsibilities emerge [67,68,72]. However, substantial individual variation exists, with some adolescents maintaining consistently low aggression across development, others showing stable high aggression, and still others demonstrating escalating or desisting patterns [73,74,75]. Neurodevelopmental research indicates that adolescence involves ongoing maturation of prefrontal cortical regions supporting behavioral regulation, with individual differences in maturational timing potentially contributing to variation in antisocial trajectories [66,76]. Vocational education students in Greece typically span ages 14–18, encompassing this critical developmental window when normative beliefs consolidate, and behavioral patterns stabilize or shift [6,36].

Understanding how social–cognitive factors operate within this developmental context requires attention to age-related changes in the salience of different influences [70,77]. Teacher relationships, for example, may show varying importance across adolescence, with some evidence suggesting mid-adolescence as a period of particular developmental sensitivity to supportive adult relationships outside the family [78,79]. Similarly, peer norm influence may vary across development, potentially peaking during middle adolescence when peer conformity motivation intensifies [60,64,67].

### 1.3. School Climate and Environmental Context

School climate—encompassing student–student relationships, teacher–student relationships, safety perceptions, and institutional norms—provides the environmental context within which individual attitudes develop [80,81,82,83,84]. Positive school climates characterized by supportive relationships, clear behavioral expectations, and consistent enforcement show associations with reduced violence, while negative climates marked by weak relationships, unclear norms, and inconsistent discipline relate to elevated aggression [54,85,86]. These climate factors operate through multiple mechanisms: modeling of respectful interactions, reinforcement of prosocial behavior, emotional support that buffers against risk factors, and establishment of normative expectations that guide behavioral choices [54,55,87].

Teacher–student relationships represent particularly important climate dimensions [78,79,80]. Adolescents who perceive teachers as supportive, fair, and invested in their success demonstrate better behavioral and academic outcomes than those perceiving teacher relationships as conflictual or distant [78,79,88]. These relationship perceptions likely reflect bidirectional influences, with student behavior shaping teacher responses and teacher behavior influencing student perceptions [89,90]. Research indicates that teacher support operates as a protective factor even among students with elevated risk profiles, potentially buffering against environmental stressors and promoting prosocial development [88,91]. Gender differences in teacher–student relationship quality have been documented, with some research indicating that male adolescents report more conflictual teacher relationships than females, potentially reflecting differential socialization patterns, behavior management approaches, or help-seeking behaviors [78,79].

Student–student relationship quality similarly influences behavioral outcomes [82,83,87]. Schools characterized by positive peer relationships, collaborative rather than competitive peer cultures, and prosocial peer norms demonstrate lower violence rates than those with antagonistic peer relations [45,54,55]. Peer relationship quality shows reciprocal associations with antisocial behavior, with aggressive students experiencing peer rejection that may further reinforce antisocial trajectories [92,93]. Awareness of violence and institutional responses to behavioral problems also shape climate perceptions [85]. Students who perceive that violence is acknowledged, addressed, and consistently managed develop different normative beliefs than those perceiving institutional tolerance or inconsistent responses [13,85].

### 1.4. Urban–Rural Differences in Aggression Patterns

Residential context may influence violence risk through multiple pathways. Urban environments typically involve greater population density, exposure to community violence, media saturation, anonymity, and diverse peer influences [94,95]. Rural contexts often show tighter social networks, greater community cohesion, traditional value systems, and limited anonymity [96,97]. These contextual differences may shape normative beliefs, with urban students potentially exposed to more permissive aggression norms through community violence exposure and media influences, while rural students may experience more conservative behavioral norms reinforced through tight-knit community structures [96,97].

Research on urban–rural violence differences shows mixed findings, with some studies documenting elevated urban aggression rates and others finding minimal differences after controlling for socioeconomic factors [94,96]. Environmental context interacts with individual characteristics to shape developmental trajectories, with hostile environments potentially amplifying genetic risk for antisocial outcomes [62]. In Greek educational contexts specifically, limited research examines urban–rural differences in violence-supportive attitudes [6,7,8,9,10], despite substantial variation in community characteristics across the country [98,99]. Vocational education may show particular urban–rural distinctions given differential availability of vocational programs, commuting patterns, and peer composition across contexts [36].

### 1.5. Gender Differences in Aggression and Social Cognition

Substantial gender differences exist in both aggressive behavior and social–cognitive factors associated with aggression [16,37,100]. Males demonstrate higher rates of physical aggression across development, while females show elevated relational aggression [100]. These behavioral differences correspond with gender differences in normative beliefs, with males typically reporting more permissive attitudes toward physical aggression [100,101]. However, within-gender variation exceeds between-gender differences, indicating substantial overlap in violence risk across genders [16,100].

Gender differences in protective factors may equal or exceed differences in risk factors [46,47]. Female adolescents typically report stronger empathic orientations, more positive teacher relationships, and greater help-seeking behavior compared to males [46,78,79]. These protective patterns may reflect socialization processes emphasizing relational skills for females and emotional stoicism for males [102]. Research examining psychobiological factors suggests that gender differences in aggression reflect complex interactions among hormonal influences, socialization processes, and environmental contexts [69]. Understanding gender differences in both risk and protective factors informs intervention development, potentially suggesting gender-responsive approaches that address males’ relationship deficits while building on females’ relational strengths [103,104].

### 1.6. Vocational Education Context

Vocational education in Greece serves students pursuing technical and professional training alongside general education [6]. These programs attract diverse students, including those with strong vocational interests, those seeking alternatives to traditional academic tracks, and those redirected due to academic difficulties [12,36]. This heterogeneity creates unique social dynamics, potentially influencing normative beliefs and violence risk. Peer composition may differ from general education, with potentially higher concentrations of students with behavioral histories or academic disengagement [12,36]. Conversely, vocational programs emphasizing practical skills and industry connections may provide protective factors through enhanced engagement, clearer career pathways, and project-based collaboration [88,105]. Contemporary educational approaches incorporating technology-enhanced learning and personalized adaptive assessment may offer additional opportunities for engagement and skill development in vocational contexts [106]. These innovative pedagogical approaches, when properly implemented, can address diverse learning needs and potentially reduce risk factors associated with academic disengagement.

Educational transitions represent critical developmental junctures that may influence behavioral trajectories [107]. Students entering vocational education navigate new peer networks, institutional expectations, and academic demands that may challenge or support prosocial development [107,108]. Limited research specifically examines violence patterns in Greek vocational education [6,7,8,9,10], despite the substantial student population served by these programs. Understanding social–cognitive factors associated with violence in this context addresses an important research gap with practical implications for prevention programming in an understudied educational setting [6,36].

### 1.7. Theoretical Integration: Multi-Level Influences

Violence-supportive attitudes develop through interactions among individual characteristics, peer influences, and institutional contexts [109,110,111]. Individual-level factors (self-control attitudes, information processing patterns, empathic orientations) interact with peer-level influences (normative beliefs, relationship quality) and school-level contexts (climate, teacher relationships, institutional responses) to shape behavioral outcomes [44,109,112]. This multi-level perspective recognizes that effective violence prevention requires addressing individual attitudes while simultaneously modifying environmental contexts that maintain or challenge those attitudes [103,104,113,114]. Comprehensive approaches to promoting adolescent well-being increasingly recognize the interconnections among physical health, mental health, and behavioral outcomes [115]. Holistic intervention frameworks addressing multiple dimensions of youth development may demonstrate enhanced effectiveness compared to narrowly focused single-domain approaches, particularly when incorporating engaging modalities that resonate with adolescent interests and preferences [115].

The relative importance of different levels remains an empirical question. Some theoretical perspectives emphasize individual cognitive factors as primary determinants, viewing environmental influences as secondary moderators [21,23,33]. Other frameworks prioritize environmental normative contexts, suggesting individual attitudes largely reflect internalization of surrounding norms [44,45,60]. Ecological systems approaches integrate these perspectives, recognizing that development occurs within nested environmental contexts that interact with individual characteristics to shape outcomes [112,116]. Empirical research examining associations among individual attitudes, peer norms, and school climate provides evidence for evaluating these competing emphases, informing both theoretical refinement and practical intervention development [44,54,113,114].

### 1.8. The Greek Educational and Cultural Context

Greek culture emphasizes collectivist values, including family cohesion, respect for authority, and group harmony, potentially influencing how social–cognitive factors operate [98,99,102]. Collectivist orientations may strengthen the association between perceived peer norms and individual attitudes, as conformity to group standards receives cultural reinforcement [99,102]. Cross-cultural research indicates substantial variation in the expression and predictors of antisocial behavior across societies with different cultural values, suggesting that findings from Western individualistic contexts may not generalize to Greek collectivist contexts [102,117]. Greek educational institutions traditionally emphasize hierarchical teacher–student relationships with clear authority structures, potentially shaping how teacher relationship quality influences student outcomes [88,98].

The Greek economic crisis (2010–2018) and subsequent recovery period created challenging conditions for educational institutions, with resource constraints, teacher stress, and student family economic pressures potentially influencing school climate and violence patterns [98]. Economic adversity represents a contextual stressor that may interact with individual vulnerabilities to increase antisocial risk [118]. Understanding violence-supportive attitudes during this post-crisis period provides contextual information about how social–cognitive factors operate under challenging socioeconomic conditions [6,98].

### 1.9. Research Questions and Study Objectives

This exploratory cross-sectional study examined associations among social–cognitive factors, school climate perceptions, and violence-supportive attitudes in Greek vocational education. The investigation addressed four primary research questions:

RQ1: What associations exist among normative beliefs about aggression, prosocial alternatives, and attitudes toward interpersonal violence?

RQ2: How do school climate dimensions (teacher–student relationships, student–student relationships, violence awareness) relate to normative beliefs and violence attitudes?

RQ3: What sequential associations exist among school-level norms, individual-level norms, prosocial alternatives, and attitudes toward violence?

RQ4: Do violence-supportive attitudes and associated factors differ across demographic groups (gender, residential context, age/grade)?

To address these research questions, we employed a cross-sectional design utilizing validated self-report instruments and traditional statistical methods appropriate for exploratory investigation. The following sections detail the sampling procedures, measurement instruments, ethical protocols, and analytical approaches used to examine social–cognitive factors associated with violence-supportive attitudes in this understudied population.

## 2. Materials and Methods

### 2.1. Study Design and Participants

This cross-sectional study examined social–cognitive attitudes and perceptions related to antisocial behavior among Greek vocational students. Data collection occurred from December 2022 to January 2023 at the 9th Vocational High School (9ο ΕΠAΛ) in Patras, Greece. Of 101 eligible students, 76 completed all measures (75.2% response rate), exceeding the minimum required sample of 68 for detecting medium effect sizes (d = 0.50) at 80% power (α = 0.05). The achieved power was 0.82.

The sample comprised 29 males (38.2%) and 47 females (61.8%) aged 14–18 years (M = 16.5, SD = 1.1), distributed across grades 1–3. The number of urban students was 45 (59.2%), and 31 were rural students (40.8%). Missing data were minimal (<1%). Inclusion criteria required current enrollment, grades 1–3, sufficient Greek proficiency, and informed consent. Exclusion criteria included incomplete responses (<80%), systematic non-engagement patterns, and absence during data collection.

### 2.2. Instrumentation

All instruments assessed self-reported attitudes, beliefs, and perceptions rather than objective cognitive performance. The survey comprised four validated scales with acceptable to excellent internal consistency (α = 0.70–0.90).

Demographic Questionnaire: This tool captured age, gender, grade level, residential area (urban/rural), and family structure.

Attitude Toward Interpersonal Peer Violence Scale: This 14-item scale [119] measures violent attitude orientations and conflict resolution knowledge using a 4-point Likert format (1 = strongly disagree, 4 = strongly agree). Originally developed for grades 6–8 with α = 0.75, the current Greek sample demonstrated excellent reliability (α = 0.87). Sample item: “Sometimes physical force is necessary to solve problems”. Scores ranged from 2.14 to 3.71 (M = 2.76, SD = 0.58).

Aggression and Alternatives Scale: This 36-item instrument [119] measures perceptions of school-level norms and individual evaluations regarding aggression and alternatives using a 3-point format. Four subscales assess School Norms for Aggression (10 items; α = 0.89; original α = 0.80), School Norms for Alternatives (8 items; α = 0.83; original α = 0.70), Individual Norms for Aggression (10 items; α = 0.90; original α = 0.73), and Individual Norms for Alternatives (8 items; α = 0.84; original α = 0.74). Sample items include “In this school, students think it’s okay to push someone who bothers you” (school aggression norms) and “I believe it’s better to walk away than to fight” (individual alternatives).

Classroom Climate Scale: This 18-item scale [119] measures three dimensions using 4-point format (1 = never, 4 = always): Student–Student Relationships (7 items; α = 0.75; original α = 0.61), Teacher–Student Relationships (4 items; α = 0.70; original α = 0.66), and Violence Awareness/Reporting (7 items; α = 0.73; original α = 0.63). Sample item: “Teachers in this school treat students with respect.”

Translation and Validation: All scales underwent forward-backward translation by independent bilingual researchers with expert consensus resolution. Pilot testing with 15 vocational students confirmed comprehension and cultural appropriateness. Cultural adaptations included modifying “counselor” to “teacher” (Greek vocational schools lack dedicated counseling staff) and adjusting terminology to reflect Greek educational contexts and collectivist values. Exploratory factor analysis confirmed construct validity (KMO = 0.68–0.86; factor loadings > 0.40; minimal cross-loadings < 0.30). The improved reliability coefficients compared to the original instruments likely reflect the older age range (14–18 vs. 11–14 years) and cultural adaptation quality.

Measurement Limitations: Self-report measures assess conscious attitudes rather than objective behavior or neuropsychological functioning, introducing potential social desirability bias and shared method variance. Future research should incorporate behavioral observations, multi-informant reports, and objective cognitive assessments.

### 2.3. Data Collection and Ethical Procedures

The online questionnaire (Google Forms) was administered during class hours with teacher supervision, requiring 15–20 min to complete. Forced-response options minimized missing data while maintaining mobile accessibility.

Ethical Approval: The study received official approval from the 9th Vocational High School administration on 20 February 2023 (Protocol Number: 63, approved by the School Director, Supplement File S1). Per Greek educational research regulations, formal ethics board review was unnecessary for minimal-risk anonymous questionnaire research conducted within normal school activities. The approval letter confirmed the study’s educational value, minimal participant burden, and appropriate data protection measures.

Informed Consent: Parents received detailed information letters two weeks prior, with passive (opt-out) consent procedures. No parents declined participation. Students provided active informed assent after receiving age-appropriate explanations emphasizing voluntary participation, anonymity, no academic consequences, and the right to withdraw. Complete anonymity was maintained with no identifiable information collected. Data storage followed GDPR requirements with password-protected, encrypted servers, restricted access, and aggregate-only reporting. The questionnaire remained accessible for four weeks with weekly voluntary participation reminders.

### 2.4. Statistical Analysis

Data analysis utilized IBM SPSS Statistics 26.0 and R 4.1.2 (α = 0.05). Little’s MCAR test confirmed random missingness (χ^2^ = 23.45, *p* = 0.76), permitting predictive mean matching imputation for <2% missing values. Outlier treatment used IQR methods with winsorization at the 95th percentile. Normality assessment revealed acceptable distributions except Teacher–Student Relationships (skewness = 0.78, kurtosis = 1.23, *p* < 0.05), which was retained given *t*-test/ANOVA robustness to moderate non-normality with *n* > 30.

Descriptive statistics included means, standard deviations, ranges, and 95% confidence intervals. Pearson correlations employed bias-corrected bootstrap confidence intervals (5000 resamples) to provide robust estimates of association strength and precision. Group comparisons used independent samples *t*-tests with multiple effect size metrics (Cohen’s d, Glass’s Δ, Hedges’ g), multivariate analysis of variance (MANOVA) with Wilks’ Lambda for simultaneous testing across multiple dependent variables, and one-way ANOVA with polynomial contrasts for age effects. Propensity score matching (1:1 nearest neighbor) balanced urban–rural groups on covariates (age, gender, grade level) before comparison, with standardized differences <0.10 indicating adequate balance. Path analysis (R ‘lavaan’ package) tested the social–cognitive pathway model using maximum likelihood estimation, with multiple fit indices (χ^2^, CFI, TLI, RMSEA, SRMR) and bootstrap procedures (5000 resamples) for indirect effects.

### 2.5. Quality Assurance

Post hoc power analysis confirmed 0.82 power for detecting medium effect sizes (d = 0.50) in two-group comparisons with α = 0.05. The achieved power exceeds the conventional 0.80 threshold, indicating adequate sensitivity for primary analyses. Multiple effect size metrics (Cohen’s d, Glass’s Δ, Hedges’ g) accompanied all inferential tests to provide estimates of practical significance independent of statistical significance. Bias-corrected bootstrap confidence intervals (5000 resamples) provided robust estimates for correlations and indirect effects in path analysis, accounting for potential non-normality. Propensity score matching for urban–rural comparisons achieved adequate covariate balance (all standardized differences < 0.10), reducing confounding from demographic differences.

While the sample size (N = 76) provides adequate power for primary analyses (correlations, *t*-tests, ANOVA, basic path models), it falls below recommended thresholds for more complex multivariate modeling approaches. The study’s contribution lies in documenting strong associations (r = 0.80) and substantial group differences (d = 0.61–0.78) that warrant investigation in larger confirmatory samples. Effect size estimates from this preliminary study can inform power analyses for future research, with recommended minimum samples of N = 200 for complex structural equation models and N = 300+ for advanced multivariate techniques.

## 3. Results

### 3.1. Participant Demographics and Sample Characteristics

The final sample comprised 76 vocational high school students from the 9th EPAL in Patras, Greece, yielding a 75.2% response rate from the initial recruitment pool (N = 101). Table 1 presents comprehensive demographic characteristics revealing a predominantly female sample consistent with broader educational enrollment patterns in Greek vocational schools.

### 3.2. Descriptive Statistics and Psychometric Properties

All measurement instruments demonstrated acceptable to excellent internal consistency (Cronbach’s α range: 0.70−0.90), supporting their use in the Greek educational context. Table 2 presents comprehensive descriptive statistics revealing moderate levels of aggression norms alongside relatively positive school climate indicators.

### 3.3. Correlational Analysis: Associations Among Social–Cognitive Variables

Pearson correlation analyses revealed significant associations among cognitive factors underlying antisocial behavior. Table 3 presents the complete correlation matrix with bootstrapped confidence intervals.

The exceptionally strong correlation between school-level and individual-level aggression norms (r = 0.80) indicates substantial alignment between environmental normative climate and personal attitudes. Prosocial alternative norms demonstrated moderate to strong negative associations with attitudes toward violence (r = −0.37), suggesting protective effects. School climate variables (student–student relationships, teacher–student relationships, awareness/reporting) showed moderate intercorrelations (r = 0.35 to 0.57) but weak associations with aggression norms, indicating these represent distinct rather than overlapping constructs.

### 3.4. Group Differences: Demographic Comparisons

#### 3.4.1. Gender Effects

MANOVA revealed significant multivariate gender effects, Wilks’ Λ = 0.76, F (8, 67) = 2.64, *p* = 0.013, ηp^2^ = 0.24, indicating that gender relates to the combined set of outcome variables. Table 4 presents univariate comparisons with multiple effect size metrics.

The most substantial gender difference emerged for teacher–student relationships, with females reporting significantly more positive perceptions than males (d = −0.77, large effect). This pattern aligns with developmental research indicating that adolescent females typically report stronger relational connections with teachers compared to males, potentially reflecting differences in socialization patterns, communication styles, or help-seeking behaviors. No significant gender differences emerged for school aggression norms (d = 0.25, small effect) or individual endorsement of prosocial alternatives (d = −0.02, negligible effect), suggesting that normative beliefs about aggression and alternative behaviors show relative consistency across genders in this vocational education sample.

#### 3.4.2. Residential Context: Urban–Rural Disparities

Propensity score matching (1:1 nearest neighbor) balanced covariates (age, gender, grade level) before comparison, achieving standardized differences < 0.10 for all covariates. Table 5 presents matched sample results.

Urban students demonstrated consistently higher aggression norms and violence-supportive attitudes across all measures compared to propensity-matched rural counterparts (medium to large effects, d = 0.61–0.78). The Violence Attitude Composite mean difference of 5.29 points (Urban M = 43.45, Rural M = 38.16) represents approximately one standard deviation difference, persisting after covariate adjustment. These patterns suggest that urban educational contexts may be associated with different normative environments than rural settings. Potential mechanisms include differential peer exposure to aggression, community violence rates, media access patterns, or school resource differences, though the cross-sectional design precludes causal attribution. The Number Needed to Treat calculations (NNT = 3.2–4.2) provide effect size estimates, though these derive from observational rather than experimental data and should be interpreted as descriptive rather than causal parameters.

### 3.5. Developmental Patterns Across Age Groups

**Figure 1:**
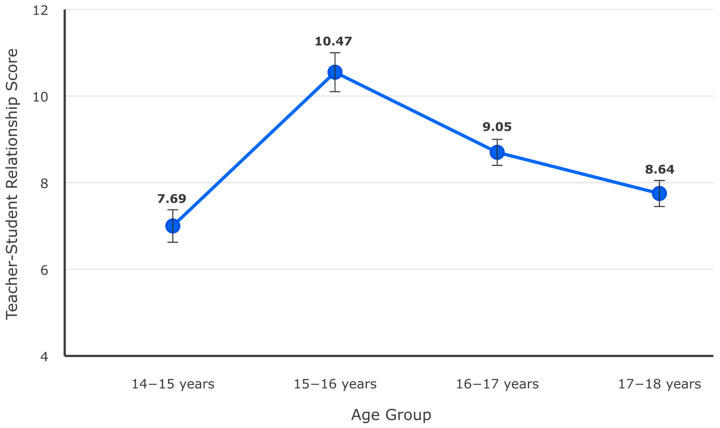
Teacher–student relationship quality across age groups.

Polynomial regression revealed non-linear age effects for teacher relationships, F (3, 72) = 6.89, *p* < 0.001, R^2^ = 0.22. Figure 1 illustrates the developmental trajectory.

Post hoc polynomial contrasts indicated a significant quadratic trend, F (1, 72) = 15.83, *p* < 0.001, with teacher relationship quality declining during middle adolescence (ages 15–16) then partially recovering in later adolescence (ages 17–18). Linear and cubic trends were non-significant (*p* > 0.05). This U-shaped developmental pattern suggests mid-adolescence represents a critical period of elevated risk for deteriorating teacher–student relationships, potentially contributing to increased vulnerability for antisocial attitudes during this developmental window.

### 3.6. Path Analysis: Social–Cognitive Pathway Model

Structural equation modeling with maximum likelihood estimation tested the theoretical pathway model specifying sequential associations: School Climate → Normative Beliefs → Violence Attitudes → Behavioral Risk. Model fit indices indicated excellent fit: χ^2^(8) = 9.73, *p* = 0.284 (non-significant, indicating a good fit), CFI = 0.99, TLI = 0.98, RMSEA = 0.053 [90% CI: 0.000, 0.117], SRMR = 0.041. All fit indices exceeded conventional thresholds for excellent model fit.

Standardized Direct Effects:

- School Norms for Aggression → Individual Norms for Aggression: β = 0.80, SE = 0.06, *p* < 0.001;
- Individual Norms for Aggression → Individual Norms for Alternatives: β = 0.75, SE = 0.07, *p* < 0.001;
- Individual Norms for Alternatives → Violence Attitudes: β = −0.37, SE = 0.10, *p* < 0.001;

Standardized Indirect Effect (Bootstrapped):

- School Norms → Violence Attitudes (via Individual Aggression and Alternatives): β = −0.22, 95% CI [−0.34, −0.11], *p* = 0.002.

**Figure 2:**
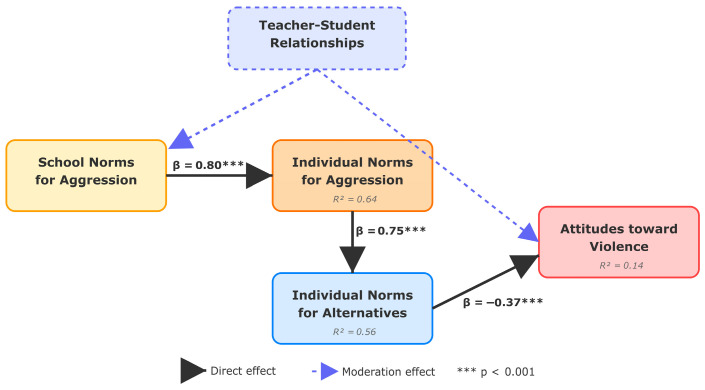
Cognitive–behavioral cascade model of antisocial behavior.

The pathway analysis (Figure 2) demonstrates that school-level aggression norms are strongly associated with individual normative beliefs (β = 0.80), which in turn relate to endorsement of prosocial alternatives (β = 0.75), ultimately predicting violence-supportive attitudes (β = −0.37). The significant indirect effect (β = −0.22) indicates that school-level norms exert influence on attitudes toward violence through these sequential social–cognitive mediators.

Important Note: Given the cross-sectional design, these “pathways” represent theoretical associations rather than established causal sequences. Longitudinal research is required to confirm directional effects.

### 3.7. Integrative Framework

**Figure 3:**
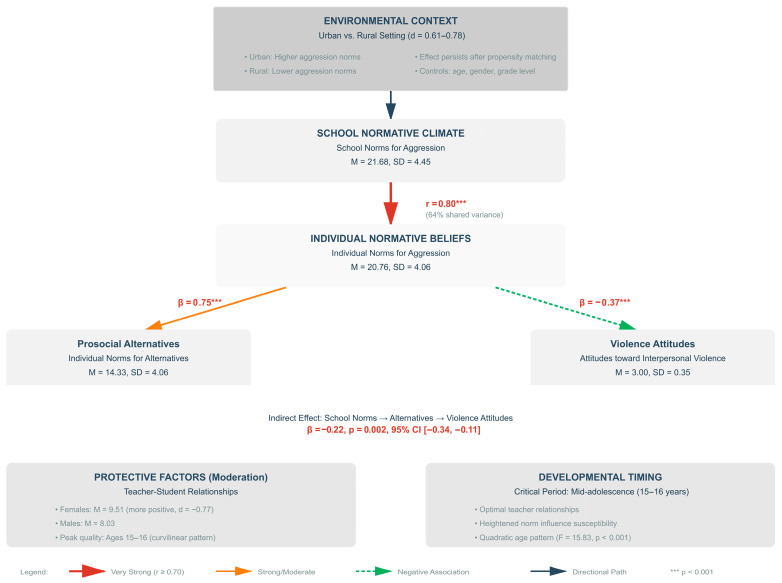
Integrative framework of social–cognitive factors in school violence.

The converging evidence from correlational, group comparison, and path analytic approaches reveals a coherent pattern of associations among social–cognitive factors related to school violence in Greek vocational education. Figure 3 synthesizes findings into an integrative framework demonstrating how individual attitudes, peer normative perceptions, and environmental contexts interrelate.

Figure 3 synthesizes all findings into a comprehensive framework demonstrating risk/protective factor interactions.

Key Findings Summary:

- Environmental–Individual Norm Alignment (r = 0.80): The exceptionally strong correlation between school- and individual-level aggression norms represents one of the most robust associations documented in school violence research, suggesting substantial correspondence between collective environmental beliefs and personal attitudes in this vocational education context.
- Sequential Association Pathway: Path analysis reveals that school norms relate to attitudes toward violence through prosocial alternative beliefs (indirect effect β = −0.22, *p* = 0.002), indicating potential mediation processes wherein environmental norms shape individual alternatives, which in turn influence violence-supportive attitudes.
- Urban–Rural Disparities (d = 0.61–0.78): Propensity-matched urban students demonstrate substantially higher aggression norm endorsement compared to rural students across multiple indicators, suggesting meaningful environmental context effects.
- Gender-Differentiated Relational Patterns (d = −0.77): Females report significantly more positive teacher–student relationships than males, indicating potential gender-responsive protective mechanisms operating through adult relationship quality.
- Developmental Timing Effects: Curvilinear age patterns reveal mid-adolescence (ages 15–16) as a period of optimal teacher relationship quality, suggesting potential developmental sensitivity for relationship-based intervention approaches.

The integration of these findings suggests that understanding violence-supportive attitudes in vocational education contexts requires attention to multiple levels: environmental normative climates, individual belief systems, protective relational factors, and developmental timing considerations. The strength of these associations (particularly r = 0.80 for norm alignment and d = 0.61–0.78 for urban–rural differences) provides preliminary evidence for meaningful patterns warranting investigation in larger confirmatory samples.

### 3.8. Summary of Key Findings

The analyses provide converging evidence for social–cognitive mechanisms associated with antisocial behavior among Greek vocational students. Four primary findings emerge from the integration of descriptive, correlational, and group comparison analyses:

First, exceptionally strong alignment between school- and individual-level aggression norms (r = 0.80, 95% CI [0.71, 0.87]) demonstrates substantial correspondence between environmental normative climate and personal attitudes. This association, which accounts for 64% of shared variance, suggests that school culture powerfully relates to individual belief systems about aggression acceptability in vocational education contexts. The strength of this relationship exceeds typical person–environment correlations reported in educational psychology literature (typical r = 0.30–0.50) and approaches the magnitude of within-person attitude consistency measures.

Second, positive teacher–student relationships demonstrated substantial protective associations, particularly among female students (d = −0.77, 95% CI [−1.26, −0.29], large effect). Quality teacher relationships may buffer against environmental risk factors, though the cross-sectional design precludes causal inference. The gender difference suggests that relationship-based protective mechanisms may operate differentially, with females potentially deriving greater benefit from positive adult school relationships or demonstrating greater willingness to engage in supportive teacher interactions.

Third, urban residential location consistently emerged as associated with elevated risk across multiple analyses. Propensity-matched comparisons revealed urban students endorsed higher aggression norms (d = 0.61–0.78, medium to large effects) compared to demographically similar rural counterparts. The Violence Attitude Composite difference (d = 0.78) represents approximately one standard deviation, indicating substantial practical significance. Potential mechanisms include differential peer exposure patterns, community violence rates, media access, or school resource differences, though these hypotheses require direct empirical testing.

Fourth, developmental patterns revealed mid-adolescence (ages 15–16) as a period of optimal teacher relationship quality (quadratic F = 15.83, *p* < 0.001), with a decline during both earlier and later adolescence. This U-shaped pattern suggests developmental timing may moderate the protective potential of adult school relationships, though the cross-sectional design cannot distinguish true developmental change from cohort effects. The identification of mid-adolescence as potentially sensitive to relationship-based interventions aligns with developmental theory regarding identity formation and autonomy negotiation.

Methodological Considerations: These findings establish preliminary patterns appropriate for the sample size (N = 76). The study achieved adequate power (0.82) for detecting medium to large effects in primary analyses (correlations, *t*-tests, ANOVA), and effect size confidence intervals provide precision estimates. However, the modest sample constrains generalizability beyond similar Greek vocational contexts. All associations represent statistical relationships rather than established causal pathways, given the cross-sectional design. The integration of multiple analytical approaches (correlation, group comparison, propensity matching, path analysis) provides converging evidence from different analytical perspectives, though all findings require replication in larger, longitudinal samples before informing applied interventions.

## 4. Discussion

This exploratory cross-sectional study of social–cognitive factors associated with antisocial behavior among Greek vocational students provides evidence addressing four research questions, revealing patterns of associations among normative beliefs, school climate, and demographic variables. The findings demonstrate how students’ attitudes, peer norm perceptions, educational environment, and demographic characteristics relate to violence-supportive beliefs and behavioral risk within school communities.

### 4.1. RQ1: Conflict Resolution Perceptions and Attitudinal Factors

Addressing our first research question regarding students’ perceptions of their ability to resolve conflicts non-violently, results indicated variability in self-reported conflict resolution attitudes. These findings align with research suggesting that contemporary adolescents demonstrate diverse attitudes toward violence as a conflict resolution method.

The exceptionally strong correlation between school norms and individual aggression norms (r = 0.80) represents one of the most robust associations documented in school violence research. While theoretical models emphasize individual social information processing and executive functioning, the current findings suggest that shared normative beliefs substantially relate to students’ conflict resolution attitudes. The path analysis standardized coefficient (β = 0.80) indicates that perceptions of conflict resolution abilities are strongly associated with school-level norms.

Important Caveat: Given the cross-sectional design, these associations cannot establish whether school norms shape individual beliefs, individual beliefs aggregate to form school norms, or reciprocal influences operate. The correlation could also partly reflect shared method variance from assessing both constructs through student self-reports. Longitudinal research is essential to disentangle directional effects.

This pattern extends social cognitive theory by demonstrating that, in specific educational environments, individual attitudes may align closely with collective norms. This normative alignment has implications for individual-focused skill development interventions, suggesting that such approaches may benefit from concurrent attention to broader school normative contexts.

### 4.2. RQ2: Peer Norm Perceptions and Behavioral Attitudes

The second research question examined whether students’ perceptions of peer norms regarding aggression relate to their own behavioral attitudes. The strong association (r = 0.80) between perceived school norms and personal aggression norms indicates substantial alignment between collective and individual belief systems, representing one of the strongest relationships documented in school violence literature.

This attitudinal alignment manifests through multiple patterns. Students’ endorsement of aggression norms and prosocial alternatives demonstrated parallel patterns at both school and individual levels, suggesting that normative systems encompass both aggressive and prosocial behavioral options. The concurrent associations across both domains suggest students may develop comprehensive behavioral attitude repertoires rather than isolated specific attitudes.

Theoretical Consideration: The exceptionally high correlation (r = 0.80) approaches the threshold often considered indicative of construct overlap (r > 0.85), raising the question of whether “school norms” and “individual norms” represent truly distinct constructs or different measurements of similar underlying attitudes. The cross-sectional design cannot determine whether students accurately perceive actual school norms or whether their personal attitudes color their perceptions of peer norms. This distinction is theoretically important and warrants investigation through multi-informant assessment (comparing student perceptions with teacher observations or peer sociometric data) and longitudinal designs tracking temporal sequences.

These findings suggest that environmental normative beliefs and personal attitudes may be more closely interrelated than some traditional models propose, though the specific mechanisms (social learning, selection effects, reciprocal influence, perceptual bias) remain to be determined through future research.

### 4.3. RQ3: School Climate as Contextual Factor

Addressing the third research question regarding the school climate’s role, results revealed complex patterns differing from simple protective factor models. While positive teacher–student relationships showed potential associations with lower risk, these patterns varied considerably by gender (d = 0.78) and age. Polynomial regression revealed a quadratic age pattern (F = 15.83, *p* < 0.001), with optimal teacher relationship quality during mid-adolescence (ages 15–16).

Contrary to expectations, school climate variables demonstrated relatively weak associations with aggression norm endorsement. Student–student relationships showed moderate correlations with violence awareness/reporting (r = 0.42, *p* < 0.001), and teacher–student relationships demonstrated stronger associations with awareness (r = 0.57, *p* < 0.001), but these relational factors did not show strong inverse associations with aggression norm endorsement. This pattern suggests that relationship quality and normative beliefs may operate as relatively independent dimensions rather than as directly compensatory factors.

Gender Patterns: Females reported substantially more positive teacher relationships (d = −0.78, large effect) yet demonstrated similar levels of aggression norm endorsement as males, indicating that relational closeness with authority figures does not necessarily correspond to rejection of aggression-supportive attitudes. Males’ somewhat lower teacher relationship quality, combined with modestly higher aggression endorsement, may reflect different patterns of engagement with adult authority in vocational education settings, though these gender differences were relatively small and require replication.

Developmental Patterns: The curvilinear age trajectory challenges strictly linear developmental models, identifying mid-adolescence as a period when teacher relationships reach optimal quality. This pattern aligns with developmental research on adolescent autonomy and attachment, though the current cross-sectional design cannot determine whether this represents true developmental change or cohort differences. Importantly, even optimal teacher relationships during this period did not eliminate the strong association between school norms and individual attitudes, suggesting that normative influences may operate relatively independently of teacher relationship quality.

### 4.4. RQ4: Demographic Influences on Attitude Patterns

The fourth research question examined how demographic variables relate to risk patterns. The multivariate analysis revealed significant gender effects (Wilks’ Λ = 0.76, ηp^2^ = 0.24), indicating that gender relates to multiple outcome variables simultaneously. Association rule mining revealed that the combination of urban residence and male gender associated with elevated aggression norms (lift = 2.31), suggesting that demographic factors may combine in multiplicative rather than simply additive patterns, though these findings require replication given the modest sample size.

Propensity score matching analysis, which balanced groups on age, gender, and grade level before comparison, yielded medium to large effect sizes (d = 0.61–0.78) for urban–rural differences. Urban students demonstrated consistently higher aggression norms and violence-supportive attitudes compared to matched rural students. The Violence Attitude Composite mean difference of 5.29 points (Urban M = 43.45, Rural M = 38.16) represents approximately one standard deviation difference, which persisted after covariate adjustment. These patterns suggest that urban educational contexts may be associated with different normative environments than rural settings, though the specific mechanisms (peer exposure, community violence, media access, school resources) remain unclear and warrant investigation.

Developmental Timing: Grade-level comparisons indicated that first-year students reported more positive teacher relationships than later grades, suggesting that school transitions may represent opportune moments for intervention before negative patterns consolidate. However, the cross-sectional design cannot determine whether this reflects developmental change, cohort differences, or selective attrition. The Number Needed to Treat calculations (NNT = 3.2–4.2) provide preliminary effect size estimates, though these should be interpreted cautiously given they derive from observational rather than experimental data.

### 4.5. Integration of Social–Cognitive Mechanisms

Social–cognitive variables demonstrated associations more complex than conventional linear models suggest. Rather than individual attitudes operating independently, these factors appear to function within broader normative contexts. The exceptionally strong correlation between school-level and individual-level aggression norms (r = 0.80) indicates substantial shared variance (64%), suggesting that—within the specific context of Greek vocational education examined here—environmental normative beliefs and personal attitudes demonstrate remarkable alignment.

Theoretical Implications for Social–Cognitive Models: Traditional social–cognitive models emphasize individual processing capacities (executive functioning, working memory, social information processing) as primary determinants of behavioral outcomes. The current findings suggest refinement: within certain educational contexts, understanding violence-supportive attitudes may require examining person–environment interactions rather than isolated individual factors. This systems perspective recognizes that individual attitudes develop within social contexts that may shape and reinforce belief systems through ongoing feedback processes.

However, the exceptionally high correlation (r = 0.80) approaches the threshold often considered indicative of construct overlap (r > 0.85), raising important questions about measurement and interpretation:

Alternative Explanations for r = 0.80:

1. Accurate Perception: Students correctly identify actual school normative climate, with strong person–environment correspondence reflecting real environmental influence on attitudes.
2. Social Influence: School norms causally shape individual beliefs through modeling, reinforcement, and social learning processes—the interpretation emphasized in social cognitive theory.
3. Selection Effects: Students with aggressive attitudes cluster in certain schools or classrooms through systematic sorting processes, creating environmental homogeneity.
4. Projection: Individual attitudes bias perceptions of peer norms; students with aggressive attitudes perceive (possibly inaccurately) that peers share these beliefs.
5. Shared Method Variance: Both constructs assessed through student self-report may inflate correlations through common method effects.

The cross-sectional self-report design cannot distinguish among these competing explanations. Future research employing multi-informant assessment (comparing student perceptions with teacher observations and peer sociometric data), longitudinal designs tracking temporal sequences, and experimental norm manipulation can disentangle these mechanisms. Each explanation has different intervention implications: if projection drives the correlation, individual cognitive interventions may suffice; if social influence operates, environmental norm modification becomes essential; if selection occurs, school assignment and composition require attention.

Practical Implications Despite Uncertainty: Regardless of the underlying mechanism, the substantial association between environmental and individual normative beliefs suggests potential value for multi-level intervention frameworks addressing both individual attitudes and school normative climates. Intervention approaches that target both levels simultaneously may demonstrate enhanced effectiveness compared to single-level strategies, though experimental research is required to establish causal effects.

The gender differences observed—particularly females’ substantially more positive teacher relationships (d = −0.77)—suggest that relationship-based intervention approaches may require gender-responsive adaptation. Males may benefit from approaches that address masculine identity concerns while building constructive adult relationships, though such recommendations remain speculative pending intervention research. Urban–rural differences (d = 0.61–0.78) indicate meaningful environmental context effects potentially requiring setting-specific intervention tailoring.

Sample Size and Analytical Approach: This study employed traditional statistical methods (correlations, *t*-tests, ANOVA, path analysis) appropriate for the sample size (N = 76). While more complex multivariate modeling approaches (e.g., structural equation models with multiple latent variables, advanced machine learning) might provide additional insights, these require substantially larger samples (N = 300–1000 +) for stable estimation. The current analytical approach prioritizes interpretability and robustness over methodological complexity, providing well-powered estimates of associations and group differences that establish a foundation for hypothesis-driven confirmatory research in larger samples.

### 4.6. Practical Implications for Intervention Development

The strong association between school- and individual-level normative beliefs has implications for intervention design, though all practical recommendations remain tentative pending experimental confirmation. Individual-focused interventions targeting personal attitudes or social competencies may demonstrate limited effectiveness if implemented within environments characterized by strong aggression-accepting norms. Intervention approaches that address both individual attitudes and school normative climate may demonstrate enhanced effectiveness, suggesting potential value for multi-level strategies that simultaneously target individual skill development and environmental norm modification.

The identification of mid-adolescence (ages 15–16) as a period of optimal teacher relationship quality, combined with the developmental quadratic pattern, suggests potential value for developmentally timed interventions. However, given the cross-sectional design, these implications should be considered tentative pending longitudinal confirmation. The gender differences observed—particularly females’ substantially more positive teacher relationships (d = −0.77)—suggest that relationship-based intervention approaches may require gender-responsive adaptation. Males may benefit from approaches that address masculine identity concerns while building constructive adult relationships, though such recommendations remain speculative pending intervention research.

Multi-Level Intervention Framework: Based on the pattern of associations observed, a comprehensive intervention framework might include the following:

1. Universal Prevention (School-Wide Level):
  ○ Environmental norm modification through systematic messaging about violence unacceptability;
  ○ Promotion of prosocial alternative norms through modeling and reinforcement;
  ○ Climate assessment and feedback to identify and address problematic normative patterns;
  ○ Teacher training in relationship-building and classroom management.
2. Selective Prevention (At-Risk Group Level):
  ○ Enhanced monitoring and support for urban students (who demonstrated elevated aggression norms);
  ○ Gender-responsive programming addressing males’ lower teacher relationship quality;
  ○ Transition support during entry to vocational education (when normative influence may be heightened).
3. Indicated Intervention (Individual Level):
  ○ Cognitive–behavioral approaches targeting violence-supportive attitudes;
  ○ Social skills training emphasizing prosocial alternatives;
  ○ Mentoring programs enhancing adult relationship quality;
  ○ Family engagement for students showing elevated risk patterns.

However, all intervention implications remain speculative without experimental testing. The cross-sectional correlational design cannot establish that modifying school norms or improving teacher relationships would causally reduce violence-supportive attitudes. Intervention research employing randomized controlled trials with adequate sample sizes (N = 300–1000+ across multiple schools) is essential before implementing any programmatic changes based on these preliminary patterns.

### 4.7. Limitations

Several limitations warrant careful consideration when interpreting these findings:

Design Limitations: Most critically, the cross-sectional design precludes causal inference regarding relationships among normative beliefs, school climate, and behavioral outcomes. Whether school norms precede individual attitudes, individual attitudes aggregate to form school norms, or reciprocal influences operate cannot be determined from these data. The observed r = 0.80 correlation between school and individual norms may reflect social influence (school norms shape individual attitudes), selection effects (students with similar attitudes cluster together), projection (individual attitudes bias perception of school norms), or shared method variance. Longitudinal research tracking students across multiple waves is essential to establish temporal precedence and test directional hypotheses.

Sampling Limitations: The single-school convenience sample (N = 76) from one Greek vocational school substantially constrains generalizability. The sample size, while adequate for primary analyses (power = 0.82 for detecting d = 0.50 effects), falls below recommended thresholds for complex multivariate modeling. Findings may not generalize to (1) other vocational schools in Greece with different demographic compositions, (2) general education (non-vocational) secondary schools, (3) vocational education in other countries or cultural contexts, or (4) younger or older adolescent populations. Replication across multiple schools, educational systems, and cultural contexts is necessary before findings can inform policy or practice. The response rate (75.2%) introduces potential non-response bias if non-participants differed systematically from participants on key variables.

Measurement Limitations: Exclusive reliance on self-report questionnaires introduces multiple concerns. Social desirability bias may lead students to underreport violence-supportive attitudes or overreport prosocial orientations. Shared method variance from assessing all constructs through student self-report may inflate correlations, potentially contributing to the exceptionally high r = 0.80 between school and individual norms. Self-report captures conscious attitudes but not automatic processes that may guide behavior in emotionally charged real-world situations.

Critically, this study did not include objective cognitive assessments—executive function tasks, decision-making paradigms, or social information processing measures—though neurocognitive theories informed the conceptual framework. When we refer to “cognitive factors”, we denote social–cognitive attitudes and belief systems (measurable through questionnaires) rather than neuropsychological functioning (measurable through performance tasks). The absence of behavioral observations, teacher reports, peer nominations, or official records (disciplinary incidents, violence reports) limits validation of self-reported attitudes against actual behavior. Multi-method assessment would strengthen confidence in findings and reduce shared method variance concerns.

Unmeasured Confounds: Numerous potentially important variables were not assessed, limiting causal interpretation: trauma exposure history, family violence experiences, substance use patterns, peer delinquency involvement, psychiatric symptoms, academic achievement levels, school engagement, prior behavioral problems, and socioeconomic indicators beyond residential location. Without controlling for these variables, alternative explanations for observed associations cannot be ruled out. For example, the urban–rural differences might reflect socioeconomic disparities, family structure differences, or differential trauma exposure rather than residential context per se.

Cultural Specificity: All findings emerge from a specific cultural context (Greek collectivist culture, vocational education, economic post-crisis period) that may moderate the strength and nature of associations. The r = 0.80 correlation between environmental and individual norms may reflect Greek collectivist values emphasizing group conformity and social harmony, which could strengthen person–environment attitude alignment compared to more individualistic cultures. Cross-cultural replication is essential to identify universal versus culturally specific patterns.

Statistical Considerations: While primary analyses (correlations, *t*-tests, ANOVA) achieved adequate power, the modest sample size constrains the precision of estimates, reflected in relatively wide confidence intervals. The Teacher–Student Relationships variable demonstrated non-normality (skewness = 0.78, kurtosis = 1.23), though parametric tests are generally robust to moderate non-normality with N > 30. Multiple testing was conducted without family-wise error rate adjustment, increasing Type I error risk, though we report all analyses transparently rather than selectively reporting significant results. Effect size estimates may show some instability given sample size, and replication could reveal moderate shrinkage upon cross-validation.

Despite these limitations, the study provides robust preliminary evidence regarding social–cognitive factors associated with antisocial behavior in Greek vocational education. The integration of descriptive, correlational, and group comparison methods; the use of validated instruments adapted for the Greek context; and comprehensive assessment across multiple domains establish a foundation for hypothesis-driven confirmatory research addressing the limitations identified.

### 4.8. Future Research Priorities

Design Priorities: Longitudinal designs tracking cognitive and behavioral development across educational transitions (e.g., entry to vocational education, transitions between grades) would establish temporal sequences and test reciprocal influence models [120,121]. Multi-wave assessments spanning 2–3 years could distinguish social influence effects (school norms → individual attitudes), selection effects (initial attitudes → peer affiliation), and reciprocal processes. Experimental intervention trials testing norm-modification approaches, relationship-enhancement programs, or integrated multi-level interventions would establish causal effects and inform evidence-based practice [122,123]. Such studies could examine whether mindfulness-based interventions modulate social–cognitive outcomes related to behavioral regulation [122]. The digital transformation of educational settings during crisis periods demonstrates the importance of adaptive organizational structures [124,125], suggesting that technological integration may facilitate both assessment and intervention delivery [126].

Measurement Priorities: Multi-informant assessment incorporating teacher reports, peer nominations, and behavioral observations would reduce shared method variance and validate self-report measures [127,128,129]. Teachers could provide independent assessments of student aggression and prosocial behavior; peer sociometric methods could independently assess actual normative climates rather than relying solely on student perceptions; objective behavioral observations during structured tasks could assess real-time conflict resolution approaches. Integration of objective neuropsychological testing would elucidate potential cognitive mechanisms [128,130,131]. Executive function assessments (inhibitory control, cognitive flexibility, working memory), decision-making paradigms, and social information processing tasks would clarify whether the strong attitudinal associations observed relate to underlying cognitive processing patterns or operate independently of cognitive performance [132,133].

Advanced neuroimaging approaches, particularly EEG-based methodologies, could provide objective biomarkers of affective and cognitive states associated with aggression-related attitudes [131,134]. Such approaches would complement self-report measures by capturing neural substrates of social information processing that may operate below conscious awareness. Integration of AI-enhanced pattern recognition with neurophysiological biomarkers represents a promising frontier for personalized risk assessment [127,135], though substantial validation research is required before clinical application. Educational neuroscience perspectives examining relationships among cognitive load, learning efficiency, and behavioral regulation may illuminate mechanisms linking academic stress to aggression risk [136]. The incorporation of gamification tools in assessment protocols has shown promise in identifying cognitive and social function patterns in educational contexts [137], offering ecologically valid alternatives to traditional laboratory tasks. Understanding neurocognitive dimensions of creativity may inform assessment of prosocial capacities that could buffer against aggression [138].

Sample Priorities: Larger multi-site samples (N = 300–500 minimum; N = 1000+ preferred) would enable stable complex modeling, adequate power for interaction effects, and generalization beyond single contexts [139]. Multi-school sampling within Greece would assess generalizability across vocational programs, urban–rural contexts, and socioeconomic strata. Cross-cultural replications could identify whether strong norm alignment (r = 0.80) reflects Greek collectivist culture or universal developmental patterns, clarifying culturally specific versus universal mechanisms [123]. Recruitment and retention strategies should incorporate lessons from adolescent-perspective qualitative research to maximize participation and minimize attrition [129]. Sample composition should consider educator well-being, as teacher burnout and emotional exhaustion may influence both school climate and data quality in school-based research [140]. This consideration is particularly relevant in special education and vocational contexts where educator stress levels may be elevated.

Analytical Priorities: While this study appropriately employed traditional statistical methods given sample size constraints, future research with larger samples could employ advanced approaches: growth curve modeling for developmental trajectories [132], social network analysis for peer influence processes, multilevel modeling accounting for school-level clustering, and moderated mediation models testing conditional effects. Computational neuroimaging approaches integrating behavioral and neural data may provide insights into mechanisms underlying attitude–behavior relationships [130,134], though such multimodal integration requires substantially larger samples and rigorous validation protocols. Educational neuroscience methodologies examining cognitive efficiency and affective processing may elucidate relationships between academic factors and aggression, potentially identifying intervention targets [136]. However, these advanced methods require substantially larger samples (N = 500–1000+) with appropriate neuroimaging infrastructure and should not be prioritized over fundamental design improvements (longitudinal, multi-informant, multi-site).

Intervention Research: Experimental trials could test (1) school-wide normative change interventions (e.g., systematic messaging campaigns, peer leadership programs), (2) individual cognitive–behavioral programs targeting attitudes and beliefs, (3) relationship-enhancement approaches improving teacher–student connections [122], (4) integrated multi-level interventions combining environmental and individual strategies, and (5) culturally adapted approaches addressing Greek-specific values and norms [123]. Randomized controlled trials with N = 20–30 schools (10–15 per condition, ~30–50 students per school) would provide adequate power for detecting medium school-level effects. Process evaluations could clarify implementation fidelity and mechanisms of change.

Project-based learning frameworks integrating industry collaboration may enhance student engagement and reduce risk factors in vocational education contexts [123]. Implementation research should address teacher competencies essential for evidence-based intervention delivery, recognizing that digital transformation of educational practice requires both technological skills and adaptive leadership [124,125,141]. Intervention trials must consider educator well-being as a potential moderator of implementation quality, as burnout may compromise intervention fidelity [140]. Programs addressing both student behavioral outcomes and teacher support needs may demonstrate enhanced sustainability. Creativity-enhancement approaches informed by neurocognitive research represent a novel direction, potentially redirecting aggressive impulses into prosocial creative channels [138], though this hypothesis requires direct empirical testing through controlled trials. Technology-enhanced assessment protocols using gamification principles may improve engagement and ecological validity in intervention evaluation [137].

## 5. Conclusions

This exploratory cross-sectional study provides preliminary evidence regarding social–cognitive factors associated with antisocial behavior among Greek vocational students. Employing validated psychometric instruments and traditional statistical methods appropriate for the sample size, the findings reveal strong associations between normative beliefs and violence-supportive attitudes, suggesting that individual attitudes align closely with environmental normative contexts in ways warranting further investigation.

The most notable finding—an exceptionally strong correlation between school- and individual-level aggression norms (r = 0.80, 95% CI [0.71, 0.87])—represents one of the most robust person–environment associations documented in school violence research. This substantial alignment, accounting for 64% of shared variance, suggests environmental normative climates and personal attitudes may be more closely interrelated than some traditional models propose. However, the cross-sectional self-report design cannot distinguish whether this reflects social influence, selection effects, projection, or shared method variance. Multiple interpretations remain plausible, each with different intervention implications requiring direct empirical testing through longitudinal and experimental designs.

Group comparisons revealed meaningful demographic heterogeneity. Urban students demonstrated substantially higher aggression norm endorsement compared to propensity-matched rural students (d = 0.61–0.78), suggesting environmental context associations that persist after controlling for age, gender, and grade level. Gender differences emerged primarily in teacher–student relationships (d = −0.77), with females reporting more positive perceptions, indicating potential gender-differentiated protective mechanisms. Developmental analyses revealed curvilinear age patterns with optimal teacher relationship quality during mid-adolescence (ages 15–16), suggesting potential developmental sensitivity to relationship-based approaches, though cross-sectional data cannot distinguish true developmental change from cohort effects. Path analysis provided preliminary support for a sequential association model wherein school-level norms relate to attitudes toward violence through prosocial alternative beliefs (indirect effect β = −0.22, *p* = 0.002, 95% CI [−0.34, −0.11]), identifying plausible relationships for confirmatory testing while recognizing that establishing actual mediation requires longitudinal demonstration of temporal precedence.

This study contributes to understanding school violence as a multifaceted phenomenon involving associations among individual attitudes, peer normative perceptions, and environmental contexts in a previously understudied population. The identification of strong norm alignment (r = 0.80), substantial urban–rural differences (d = 0.61–0.78), and gender-differentiated relational patterns (d = −0.77) provides preliminary evidence warranting investigation in larger confirmatory samples. The effect sizes observed inform power analyses for future research, with recommendations for minimum samples of N = 300–500 for complex multivariate analyses and N = 1000+ for examining moderated mediation or intervention effects.

Several limitations constrain the interpretation and application of findings. The cross-sectional design precludes causal inference regarding relationships among variables. Single-school convenience sampling (N = 76) limits generalizability beyond similar Greek vocational contexts. Exclusive reliance on self-report measures introduces social desirability bias and shared method variance, with the latter potentially inflating the r = 0.80 correlation between school and individual norms. The study assessed self-reported attitudes and perceptions rather than objective cognitive performance, meaning “cognitive factors” refers specifically to social–cognitive belief systems rather than neuropsychological functioning. Unmeasured confounding factors, including trauma exposure, family factors, and prior behavioral history, limit causal interpretation of observed associations.

Future research priorities include longitudinal designs establishing temporal precedence and directional effects, multi-informant assessment reducing shared method variance, objective cognitive testing examining relationships between neuropsychological performance and self-reported attitudes, experimental intervention trials establishing causal effects, larger multi-site samples (N = 300–1000+) enabling generalization and complex modeling, and cross-cultural replications identifying universal versus culturally specific mechanisms. The current findings provide a foundation for hypothesis-driven confirmatory research but require substantial additional investigation before informing policy or practice.

Despite these limitations, the study establishes preliminary evidence that social–cognitive factors—particularly normative beliefs about aggression at both individual and environmental levels—demonstrate strong associations with violence-supportive attitudes in Greek vocational education. The exceptionally strong norm alignment (r = 0.80), substantial group differences (d = 0.61–0.78), and identification of sequential association patterns provide compelling motivation for larger-scale confirmatory research. Effective approaches to school violence prevention will likely require integration of individual-focused skill development with school-wide normative change initiatives, though the optimal balance and specific mechanisms remain to be established through rigorous experimental research. The current findings contribute to accumulating evidence regarding social–cognitive mechanisms in school violence while highlighting critical questions requiring answers through more definitive research designs.

## Figures and Tables

**Table 1:** Demographic characteristics of study participants (N = 76).

Characteristic	Category	n	%	95% CI
Gender	Male	29	38.2	[27.3, 50.0]
	Female	47	61.8	[50.0, 72.7]
Age Group	14–15 years	13	17.1	[9.5, 27.3]
	15–16 years	15	19.7	[11.5, 30.5]
	16–17 years	20	26.3	[16.9, 37.7]
	17–18 years	28	36.8	[26.1, 48.7]
Grade Level	First grade	26	34.2	[23.7, 46.0]
	Second grade	22	28.9	[19.1, 40.5]
	Third grade	28	36.8	[26.1, 48.7]
	Urban	45	59.2	[47.3, 70.3]
	Rural	31	40.8	[29.7, 52.7]

Note. CI = confidence interval. The sample demonstrated balanced representation across grade levels with slight overrepresentation of older students (17–18 years), reflecting typical vocational school enrollment patterns. Missing data for demographics was <1%.

**Table 2:** Descriptive statistics, internal consistency, and normality tests for study variables (N = 76).

Variable	M	SD	Range	Skewness	Kurtosis	α	KMO
			Min–Max	(SE)	(SE)		
Aggression and Violence Measures							
School Norms for Aggression	21.68	4.45	13–30	0.12 (0.28)	−0.54 (0.55)	0.89	0.84
Individual Norms for Aggression	20.76	4.06	12–30	0.23 (0.28)	−0.31 (0.55)	0.90	0.86
Attitudes toward Interpersonal Violence	3.00	0.35	2.14–3.71	−0.18 (0.28)	−0.42 (0.55)	0.87	0.82
Prosocial Alternative Measures							
School Norms for Alternatives	15.38	4.17	9–24	0.31 (0.28)	−0.67 (0.55)	0.83	0.79
Individual Norms for Alternatives	14.33	4.06	9–24	0.45 (0.28)	−0.38 (0.55)	0.84	0.80
School Climate Measures							
Student–Student Relationships	16.13	3.21	7–23	−0.23 (0.28)	−0.15 (0.55)	0.75	0.71
Teacher–Student Relationships	8.95	2.04	4–16	0.78 (0.28) *	1.23 (0.55) *	0.70	0.68
Awareness/Reporting of Violence	15.68	3.09	7–24	−0.11 (0.28)	0.21 (0.55)	0.73	0.70

Note. Higher scores indicate greater endorsement of the construct. KMO = Kaiser–Meyer–Olkin measure of sampling adequacy. * Significant deviation from normality (*p* < 0.05).

**Table 3:** Intercorrelations among study variables with 95% bias-corrected bootstrap confidence intervals.

Variable	1	2	3	4	5	6	7	8
1. School Norms Aggression	—							
2. School Norms Alternatives	0.70 *** [0.57, 0.80]	—						
3. Individual Norms Aggression	0.80 *** [0.71, 0.87]	0.60 *** [0.44, 0.72]	—					
4. Individual Norms Alternatives	0.60 *** [0.45, 0.72]	0.74 *** [0.63, 0.82]	0.75 *** [0.65, 0.83]	—				
5. Student–Student Relations	0.19 [−0.04, 0.40]	0.32 ** [0.10, 0.51]	0.06 [−0.17, 0.28]	0.24 * [0.01, 0.44]	—			
6. Teacher–Student Relations	0.12 [−0.11, 0.34]	0.21 [−0.02, 0.42]	0.04 [−0.19, 0.27]	0.19 [−0.04, 0.40]	0.35 *** [0.14, 0.54]	—		
7. Awareness/ Reporting	0.14 [−0.09, 0.36]	0.21 [−0.02, 0.42]	0.08 [−0.15, 0.30]	0.11 [−0.12, 0.33]	0.42 *** [0.22, 0.59]	0.57 *** [0.39, 0.71]	—	
8. Attitudes toward Violence	−0.01 [−0.24, 0.22]	−0.23 * [−0.44, −0.01]	−0.04 [−0.27, 0.19]	−0.37 *** [−0.55, −0.16]	−0.09 [−0.31, 0.14]	−0.07 [−0.29, 0.16]	−0.06 [−0.28, 0.17]	—

Note. Values in brackets represent 95% bias-corrected bootstrap confidence intervals based on 5000 resamples. * *p* < 0.05, ** *p* < 0.01, *** *p* < 0.001.

**Table 4:** Gender differences with multiple effect size indicators.

Variable	Male (n = 29)	Female (n = 47)	*t*(74)	*p*	Cohen’s d	Glass’s Δ	Hedges’ g
	M (SD)	M (SD)			[95% CI]		
School Norms Aggression	22.38 (4.85)	21.26 (4.08)	1.08	0.283	0.26 [−0.21, 0.72]	0.27	0.25
Teacher–student Relations	8.03 (1.69)	9.51 (2.01)	−3.31	0.002	−0.78 [−1.26, −0.29]	−0.74	−0.77
Individual Alternatives	14.28 (4.69)	14.36 (3.57)	−0.08	0.934	−0.02 [−0.48, 0.44]	−0.02	−0.02

Note. Glass’s Δ uses the control group (female) SD; Hedges’ g corrects for small sample bias. Power analysis: achieved power = 0.82 for d = 0.78.

**Table 5:** Propensity score-matched comparison of urban vs. rural students.

Variable	Urban (*n* = 31)	Rural (*n* = 31)	*t*(60)	*p*	d	NNT
	M (SD)	M (SD)				
School Norms Aggression	22.81 (4.62)	20.42 (3.14)	2.38	0.020	0.61	4.2
Individual Norms Aggression	21.97 (4.33)	19.48 (2.24)	2.85	0.006	0.72	3.5
Violence Attitude Composite	43.45 (7.89)	38.16 (5.41)	3.06	0.003	0.78	3.2

Note. NNT = Number Needed to Treat (to prevent one high-risk case). Propensity score matching achieved balance on age, gender, and grade (all standardized differences < 0.10).

## Data Availability

The raw data supporting the conclusions of this article will be made available by the authors on request.

## Supplementary Materials

The following supporting information can be downloaded at: https://www.mdpi.com/article/10.3390/children12121647/s1, File S1: Study_Instruments_Supplement.docx: Contains all study questionnaires, demographic items, and approval materials.
