# Supplementary material for: Social–Cognitive Factors in Antisocial Behavior and School Violence: A Cross-Sectional Analysis of Greek Vocational Students
Source: Children (Basel). 2025 Dec 4;12(12):1647. doi: 10.3390/children12121647 (PMC12731748; doi:10.3390/children12121647)
Supplement: Supplementary file 1 [file children-12-01647-s001.zip › children-3961665-supplementary.pdf]

APPENDIX A

STUDY QUESTIONNAIRE

QUESTIONNAIRE

Demographic Data

Gender

A. Boy

B. Girl

Age

14-15

15-16

16-17

17-18

What grade are you in this year?

First year of high school

Second year of high school

Third year of high school

Place of residence

- Urban area
- Rural Area

I live at home:

with my parents

with my father

with my mother

with my grandparents

with other relatives

My parents work:

Both

Only my father

Only my mother

Neither of them works

Father's educational level:

Secondary education

University education

Master's Degree

Doctorate

Mother's Educational Level:

Secondary education

University Education

Master's Degree

Doctorate

Father's Employment

Civil Servant

Private Employee

Freelancer

Unemployed

Retired

Mother's Employment

Public Employee

Private Employee

Freelancer

Unemployed

Retired

Questionnaire 1

A. Attitude Toward School Violence

[A8. Attitude Toward Interpersonal Peer Violence]

The following questions assess either a passive or violent attitude orientation as well as knowledge and ability to resolve conflicts in a non-violent manner. You are asked to state your opinions or feelings about conflict defined as physical fighting with pushing and hitting, not just verbal.

[A1] When I avoid a fight, I am a coward.

I completely agree

I agree

Disagree

I completely disagree

[A2] The best way to stop a fight before it starts is to stop the cause (problem) that caused it

I completely agree

I agree

Disagree

I completely disagree

[A3] Anyone who does not get involved in arguments becomes even more of a target

I completely agree

I agree

I disagree

I completely disagree

[A4] There is no need to argue as there are alternative ways of dealing with an argument

I completely agree

I agree

I disagree

I completely disagree

[A5] It is right to hit someone who attacks you first

I completely agree

I agree

Disagree

I completely disagree

[A6] If my friends want to go somewhere where a fight might break out, I find it easy to say that I don't want to go with them

I completely agree

Agree

Disagree

I completely disagree

[A7] When other people's actions make me angry, I can usually deal with it without getting into a physical fight

I completely agree

Agree

Disagree

I completely disagree

[A8] If a child teases me, I usually cannot stop them unless I hit them

I completely agree

Agree

I disagree

I completely disagree

[A9] If a child at school hits me, it is more difficult for me to report it to a teacher or other adult than to hit him back

I strongly agree

I agree

Disagree

I completely disagree

[A10] If I really want to, I can stop someone from trying to pick a fight with me

I completely agree

Agree

Disagree

I completely disagree

[A11] My family would be furious with me if I got into a fight with another student, regardless of the reason

I completely agree

Agree

Disagree

I completely disagree

[A12] If a student hit me first, my family would want me to hit him back

I totally agree

I agree

Disagree

I completely disagree

[A13] I can usually tell when other students are bothering me or when I am angry

I completely agree

Agree

Disagree

I completely disagree

[A14] If other students bother me or I am angry, I do things to calm down

I totally agree

Agree

Disagree

I completely disagree

Questionnaire 2

B. Assessment of Classmates' Perceptions of Aggression and Alternative Solutions

[A12. Norms for Aggression and Alternatives]

The following questions measure students' perceptions of how other students in their schools would feel if they engaged in certain behaviors (school norms) and the students' own evaluations of these behaviors (individual approval). Please select the answer that represents you between "I like it," "I don't like it," and "I don't care."

[B1] How would you feel if a classmate at school ignored/did not care about rumors spread by other children about him/her?

I like

I dislike

I don't care

[B2] How do you think your classmates would feel if a child ignored a rumor spread by others about him/her?

I like

I dislike

I don't care

[B3] How would you feel if a classmate at school hit a child who said something bad?

I like

I dislike

I don't care

[B4] How would your classmates feel if a child hit another child who said something bad?

I like

I dislike

I don't care

[B5] How would your classmates feel if a child told another child who was starting to fight that there is a choice between fighting and other ways of solving problems?

I like

I dislike

I don't care

[B6] How would you feel if a classmate told another classmate who had started a fight that there are other ways to solve problems?

I like

I dislike

I don't care

[B7] How would you feel if a child at your school yelled at another child who said something bad?

I like

I dislike

I don't care

[B8] How do you think your classmates would feel if a child yelled at someone who said something bad?

I like

I dislike

I don't care

[B9] How would you feel if a child at your school asked a teacher or another adult for help when they were being picked on after school?

I like

I dislike

I don't care

[B10] How would your classmates feel if a child asked a teacher or another adult for help when being bullied after school?

I like

I dislike

I don't care

[B11] How would you feel if a classmate apologized to someone who accidentally pushed him/her in the classroom?

I like

I dislike

I don't care

[B12] How would your classmates feel if a child apologized to someone else who accidentally pushed them in the classroom?

The room?

I like it

I don't like it

I don't care

[B13] How would you feel if a classmate threatened another child who said something bad?

I like it

I dislike

I don't care

[B14] How would your classmates feel if a child threatened another child who said something bad?

I like

I dislike

I don't care

[B15] How would your classmates feel if one child told another child to "stop and calm down" when they started fighting?

I like

I dislike

I don't care

[B16] How would your classmates feel if one child told another to "stop and calm down" when they started fighting?

I like

I dislike

I don't care

[B17] How would you feel if a classmate hit someone else who started hitting first?

I like

I dislike

I don't care

[B18] How would your classmates feel if a child hit someone else who started hitting first?

I like

I dislike

I don't care

[B19] How would you feel if a classmate hit someone else for no reason?

I like

I dislike

I don't care

[B20] How would your classmates feel if a child hit another child for no reason?

I like

I dislike

I don't care

[B21] How would you feel if a classmate threatened another classmate because he shouted first?

I like

I dislike

I don't care

[B22] How would your classmates feel if a child threatened another child because he shouted first?

I like

I dislike

I don't care

[B23] How would you feel if a classmate avoided a fight by taking a different hallway to get to class?

I like

I dislike

I don't care

[B24] How would your classmates feel if a child avoided a fight by taking a different route to class?

I like

I dislike

I don't care

[B25] How would you feel if a classmate listened to only one side of a dispute between two children?

I like

I dislike

I don't care

[B26] How would your classmates feel if a child listened to only one side of a dispute between two children?

I like

I dislike

I don't care

[B27] How would you feel if a classmate yelled at someone for no reason?

I like

I dislike

I don't care

[B28] How would your classmates feel if a child yelled at another child for no reason?

I like

I dislike

I don't care

[B29] How would you feel if a classmate yelled at someone who yelled first?

I like

I dislike

I don't care

[B30] How would your classmates feel if a child yelled at someone who yelled first?

I like

I dislike

I don't care

[B31] How would you feel if one of your classmates threatened someone else for no reason?

I like

I dislike

I don't care

[B32] How would your classmates feel if a child threatened another child for no reason?

I like

I dislike

I don't care

[B33] How would you feel if a classmate threatened someone else who hit them first?

I like

I dislike

I don't care

[B34] How would your classmates feel if one child threatened another who hit him first?

I like it

I don't like it

I don't care

[B35] How would you feel if a classmate took a deep breath when he started to lose his temper?

I like

I dislike

I don't care

[B36] How would your classmates feel if a child took a deep breath when he started to lose his temper?

I like

I dislike

I don't care

Questionnaire 3

C. School Climate Assessment

[C5. Classroom Climate Scale]

The following questions measure three dimensions of students' and teachers' perceptions of the climate in the classroom with regard to interpersonal relationships: a) between classmates, b) between student and teacher. Please indicate the extent to which you agree or disagree with the following statements.

Interpersonal relationships between classmates

[C1] My classmates are polite to each other and support each other.

I completely agree

I agree

Disagree

Strongly disagree

[C2] My classmates from different social classes and races get along well.

I completely agree

Agree

Disagree

I completely disagree

[C3] My classmates stop other classmates who are unfair or disruptive.

I completely agree

Agree

Disagree

I completely disagree

[C4] My classmates get along well with each other most of the time.

I completely agree

I agree

Disagree

I completely disagree

[C5] My classmates listen to each other respectfully during class discussions.

I completely agree

Agree

Disagree

I completely disagree

[C6] My classmates make friends easily.

I completely agree

Agree

Disagree

I completely disagree

[C7] My classmates enjoy being at school.

I completely agree

Agree

Disagree

I completely disagree

Student-teacher relationships

[C8] Teachers treat students with respect.

I strongly agree

Agree

Disagree

Strongly disagree

[C9] Teachers praise students more often than they criticize them.

I completely agree

Agree

Disagree

I completely disagree

[C10] Teachers treat students fairly.

I completely agree

Agree

Disagree

I completely disagree

[C11] Teachers take time to help students resolve their differences.

I completely agree

Agree

Disagree

I completely disagree

Awareness / Reporting

[C12] Teachers feel free to ask other teachers for help if there is a problem with a student.

I completely agree

Agree

Disagree

Strongly disagree

[C13] Teachers know when students are being singled out or bullied.

I completely agree

Agree

Disagree

I completely disagree

[C14] Students are encouraged by teachers to report incidents of bullying and aggression at school.

I totally agree

Agree

Disagree

I completely disagree

[C15] Students know who to turn to for help if they have been mistreated by another student.

I completely agree

Agree

Disagree

I completely disagree

[C16] Students report it when one student hits another.

I totally agree

I agree

Disagree

I completely disagree

[C17] Teachers take action to solve the problem when students report bullying.

I totally agree

Agree

Disagree

I completely disagree

[C18] Students report it when one student teases or makes fun of another student.

I totally agree

Agree

Disagree

I completely disagree

APPENDIX B

STUDY APPROVAL PERMIT

ΕΛΛΗΝΙΚΗ ΔΗΜΟΚΡΑΤΙΑ
ΥΠΟΥΡΓΕΙΟ ΠΑΙΔΕΙΑΣ, ΚΑΙ
ΘΡΗΣΚΕΥΜΑΤΩΝ

ΠΕΡΙΦΕΡΕΙΑΚΗ ΔΙΕΥΘΥΝΣΗ Π/ΘΜΙΑΣ & Δ/ΘΜΙΑΣ
ΕΚΠΑΙΔΕΥΣΗΣ ΔΥΤΙΚΗΣ ΕΛΛΑΔΑΣ
ΔΙΕΥΘΥΝΣΗ Δ/ΘΜΙΑΣ ΕΚΠ/ΣΗΣ ΑΧΑΪΑΣ
9ο ΕΣΠΕΡΙΝΟ ΕΠΑ.Λ. ΠΑΤΡΑΣ

Ταχ. Δ/νση: Γεωρ. Σεφέρη 129
Τ.Κ. – Πόλη: 263 35 Πάτρα
Πληροφορίες: κ. Ριζόπουλος Α.
Τηλέφωνο: 2610643109
Fax: 2610643137
E-mail:mail@9epal-esp-patras.ach.sch.gr

Αρ. Πρωτ.:63
Πάτρα, 20 / 02 / 2023

Προς: Πανεπιστήμιο Πατρών
Τμήμα: Διοικητικής Επιστήμης και Τεχνολογίας
Υπόψη: Κας Σωτηρίας-Ήρας Αντωνοπούλου

Θέμα: «Βεβαίωση διαμοιρασμού ερωτηματολογίων στο πλαίσιο Διπλωματικής Εργασίας»
Σχετ.:

Με το παρόν έγγραφο, βεβαιώνουμε ότι, η Πετροπούλου Αναστασία, με ΑΜ (αριθμός μητρώου φοιτητή) : 1096727, του Πανεπιστήμιου Πατρών, του τμήματος : Διοικητικής Επιστήμης και Τεχνολογίας, στο πλαίσιο του Μεταπτυχιακού Προγράμματος Σπουδών: Διοίκηση Εκπαίδευσης και τίτλο διπλωματικής εργασίας: "Αποτίμηση φαινομένου σχολικής βίας: προσωπικότητα και σχολικό κλίμα ως προβλεπτικοί παράγοντες", με επιβλέπουσα καθηγήτρια, την κα. Σωτηρία-Ήρα Αντωνοπούλου, διαμοίρασε ερωτηματολόγια στο σχολείο μας και έλαβε τα αποτελέσματα που είχε θέσει ως στόχο.

Παρακαλούμε για τις δικές σας ενέργειες.

Συν.:

Ο Διευθυντής
[illegible]
Ανδρέας Αριστοτέλης Ριζόπουλος
